# Information and communications technology use among young female sex workers participating in a randomised human immunodeficiency virus prevention trial in Kampala, Uganda

**DOI:** 10.1093/inthealth/ihab013

**Published:** 2021-03-19

**Authors:** Francis X Kasujja, Hillary Mutabazi, Eva Muhanguzi, Janet Seeley, Rachel King

**Affiliations:** Social Aspects of Health Programme, MRC/UVRI and LSHTM Uganda Research Unit, Entebbe, Uganda; Social Aspects of Health Programme, MRC/UVRI and LSHTM Uganda Research Unit, Entebbe, Uganda; Institute for Global Health Sciences, University of California, San Francisco, San Francisco, CA, USA; Social Aspects of Health Programme, MRC/UVRI and LSHTM Uganda Research Unit, Entebbe, Uganda; Department of Global Health and Development, London School of Hygiene and Tropical Medicine, London, UK; Social Aspects of Health Programme, MRC/UVRI and LSHTM Uganda Research Unit, Entebbe, Uganda; Institute for Global Health Sciences, University of California, San Francisco, San Francisco, CA, USA

**Keywords:** COVID-19, developing countries, mHealth, sex workers, Uganda

## Abstract

**Background:**

This study was conducted to determine the level and feasibility of use of information and communication technology (ICT) and social media for research and service delivery among young female sex workers (YFSWs) in Kampala, Uganda.

**Methods:**

We analysed baseline data from 234 YFSWs in Kampala ages 15–24 y participating in a randomized controlled trial testing a cognitive behavioural human immunodeficiency virus prevention intervention.

**Results:**

Mobile phone ownership (68.3%) and short message service use (64.9%) were moderate and significantly lower in the 15- to 19-y age group. Computer use experience and internet access were low.

**Conclusions:**

We believe that the feasibility of ICT and social media-driven interventions among YFSWs is limited.

## Introduction

The coronavirus disease 2019 pandemic has upended medical prevention, care and research, especially studies engaged in ongoing data collection. Lockdowns, transport curbs and physical distancing have led to restrictions on in-person meetings, interrupting research activities.^1^ Many health researchers currently rely on remote visits using telehealth solutions, field-based participant monitoring and courier services to collect samples and deliver drugs. Although information and communications technology (ICT) access in low- and middle-income countries has been steadily improving, telehealth technologies can be rudimentary, as is also the case in some settings in high-income countries.^2^ A reliance on access to ICT for health research and service delivery poses a challenge to the participation of some people who are already harder to reach. Among these marginalised groups are young female sex workers (YFSWs). While internet use and mobile phone ownership in Uganda are low, at 17% and 56%, respectively, this digital divide disproportionately affects young women. Young women are about three times less likely to have ever used the internet and about half as likely to own a mobile phone compared with young men.^3^ However, the level of ICT access and use among YFSWs is largely unknown. YFSWs face a disproportionate risk of human immunodeficiency virus (HIV) and sexually transmitted infection (STI).

Female sex workers <20 y of age are up to four times more likely to acquire HIV compared with those older.^4^ This study aimed to determine the level of ICT use among YFSWs in Kampala, Uganda, with the objective of assessing whether ICT use in this population would be a feasible option for research and service delivery.

## Methods

Between January 2017 and July 2019 we collected baseline data from YFSWs in Kampala, ages 15–24 y, during a randomised trial, the Zero Transmission (ZETRA) study, testing a cognitive behavioural HIV prevention intervention. Quantitative data were collected using Audio Computer-Assisted Self-Interviews, a computer-assisted personal interviewing software, to reduce social desirability and interviewer biases and improve participants’ reporting accuracy, even among study participants who are not literate. The data were analysed using Stata version 15 (StataCorp, College Station, TX, USA). We performed χ^2^ tests of independence and Fisher's exact test to examine the association between ICT use and age group.

## Results

We interviewed 234 YFSWs with a mean age of 19.9±2.5 y. About 6.0% (n=14) had completed at least 13 y of formal education. The proportion of YFSWs who reported having some computer use experience was 40.3% (95% confidence interval [CI] 33.8 to 47.0); however, only 4.0% (95% CI 1.9 to 7.5) reported owning a computer or a tablet. In contrast, 68.3% (95% CI 61.8 to 74.4) owned a mobile phone, but only 43.0% (95% CI 35.2 to 51.1) had access to the internet via phone, tablet or computer. The internet was mainly accessed for Facebook by 51.4% (95% CI 41.4 to 61.3) and WhatsApp by 32.4% (95% CI 23.6 to 42.2). The proportion who reported using short message service (SMS) daily was 64.9% (95% CI 57.7 to 71.7). YFSWs 15–19 y of age were less likely to own a mobile phone (χ^2^[1, N=221]=8.95; p<0.00) and to use SMS (χ^2^ [1, N=191]=5.95; p=0.02) than YFSWs 20–24 y of age (Table 1).

**Table 1.** ICT use among YFSWs by age category

		Age category (years), n (%)		
Variable	Total, N (%)	15–19	20–24	p-Value
Computer use experience				
Some experience	91 (40.3)	40 (40.8)	51 (39.8)	0.88
No experience	135 (59.7)	58 (59.2)	77 (60.2)	
Computer or tablet ownership				
Owns	9 (4.0)	2 (2.0)	7 (5.6)	0.31
Does not own	215 (96.0)	96 (98.0)	119 (94.4)	
Mobile phone ownership				
Owns	151 (68.3)	54 (57.5)	97 (76.4)	<0.00*
Does not own	70 (31.7)	40 (42.6)	30 (23.6)	
Accesses internet via a phone, tablet or computer				
Can access	68 (43.0)	22 (34.5)	46 (47.9)	0.12
Cannot access	90 (57.0)	40 (64.5)	50 (52.1)	
Regular use of SMS				
Uses	124 (64.9)	44 (55)	80 (72.1)	0.02*
Does not use	67 (35.1)	36 (45)	31 (27.9)	

* Statistically significant at p<0.05.

## Discussion

These findings reveal that while the reported mobile phone access and internet use among YFSWs in Kampala are moderate, social media use is very low. On the other hand, SMS use is high, especially among YFSWs who own mobile phones. The reported education level and mobile phone ownership among YFSWs were comparable to Ugandan women in the general population.^5^ However, both education level and mobile phone ownership were much lower than that reported by females of a similar age accessing HIV services at a clinic in Kampala (51.5% and 93.7%, respectively).^6^ This suggests a mobile phone access gap for YFSWs compared with women accessing HIV services in the general population. On the other hand, internet access was similar, reflecting the generally restricted penetration of the internet in Kampala. Uganda introduced a social media tax on the use of 60 mobile social media apps, including Facebook and WhatsApp, in 2018. To access these sites, users are required to pay about US$0.05 daily, which is not affordable for low-income and other marginalised groups such as YFSWs. This could limit the reach of telehealth operational programmes and research interventions.

The disproportionate access to ICT and social media by YFSWs ages 15–19 y may limit their access to and propagation of health information within their networks. It could also make it harder to cultivate social ties and to cope.

## Conclusions

We believe that the feasibility of ICT and social media–driven interventions among YFSWs is limited. Low-tech ICT solutions may be a more practical choice for HIV telehealth interventions among YFSWs in Kampala and similar contexts. Future studies should evaluate the effectiveness of different low-tech ICT HIV prevention solutions among YFSWs in Kampala.

## Data Availability

The data underlying this article cannot be shared publicly to protect the privacy of individuals that participated in the study. The data will be shared upon reasonable request to the corresponding author.
